# Organization and dynamics of SNARE proteins in the presynaptic membrane

**DOI:** 10.3389/fphys.2015.00089

**Published:** 2015-03-19

**Authors:** Dragomir Milovanovic, Reinhard Jahn

**Affiliations:** Department of Neurobiology, Max Planck Institute for Biophysical ChemistryGöttingen, Germany

**Keywords:** SNARE proteins, clustering, membrane domains, hydrophobic interactions, ionic interactions, modularity of cluster formation

## Abstract

Our view of the lateral organization of lipids and proteins in the plasma membrane has evolved substantially in the last few decades. It is widely accepted that many, if not all, plasma membrane proteins and lipids are organized in specific domains. These domains vary widely in size, composition, and stability, and they represent platforms governing diverse cell functions. The presynaptic plasma membrane is a well-studied example of a membrane which undergoes rearrangements, especially during exo- and endocytosis. Many proteins and lipids involved in presynaptic function are known, and major efforts have been made to understand their spatial organization and dynamics. Here, we focus on the mechanisms underlying the organization of SNAREs, the key proteins of the fusion machinery, in distinct domains, and we discuss the functional significance of these clusters.

## Introduction: SNAREs as tools to understand the physical priniciples behind membrane patterning

Half a century ago biological membranes were envisioned as lipid bilayers which are covered on both faces with proteins that hydrophilically interact with the phospholipid head-groups (Robertson, 1963). However, with an increasing understanding of hydrophobic interactions between proteins and lipids (Kauzmann, 1959) it became evident that the plasma membrane is not fully covered by protein layers, but rather contains two types of proteins: the ones which span the entire bilayer (integral) and the ones which are embedded in only a single of the monolayers (peripheral). These membrane proteins were shown to undergo lateral mobility in the membrane (Lenard and Singer, 1966; Wallach and Zahler, 1968; Frye and Edidin, 1970; Singer and Nicolson, 1972). Equally important were data on the structure of lipid bilayers and on the existence of different lipid phases (i.e., solid, liquid-crystalline and liquid-ordered), as well as dependence of this phase transition on the temperature and acyl chain saturation (Steim et al., 1969; Melchior et al., 1970; Wilkins et al., 1971). Together, these developments led to a major change of concept: Membranes were considered as dynamic structures in which all components were able to diffuse laterally, with the membrane proteins “floating like icebergs in a sea” of membrane lipids (fluid mosaic model; Singer and Nicolson, 1972). This model still forms the foundation of our present understanding of biological membranes, with its major tenets being confirmed in numerous studies. However, it is becoming evident that there is an added layer of complexity in the lateral organization of proteins and lipids: Membrane contains subdomains whose properties are only slowly emerging.

The first hint for membrane subdomains resulted from experiments in which biological membranes were treated with certain detergents. Surprisingly, not all components were solubilized, i.e., incorporated individually in detergent micelles. Rather, detergent-resistant proteolipid complexes were isolated that were highly enriched in specific components. It was proposed that these complexes represent specific lipid domains in the plasma membrane, so-called lipid rafts, to which certain proteins preferentially associate (Simons and Ikonen, 1997). Certain lipid mixtures (dependent on the degree of side chain saturation and the amount of cholesterol) undergo multiple phase transitions, with equilibrium between different phases (most prominently Lo and Ld phases) coexisting at physiological temperatures. Insertion of proteins then revealed that proteins mostly partition into the Ld phase; however some proteins accumulate in the Lo-phase that is also referred as a raft (de Almeida et al., 2003, 2004). Such phase transitions were also observable in vesicles formed from blebs of the plasma membrane (Levental et al., 2011; Sezgin et al., 2012), supporting the idea that lipid phase partitioning is a major factor in generating subdomains within the membrane plane. However, single particle tracking and fluorescence correlation spectroscopy experiments suggested that the formation of such large phases is prevented in plasma membranes. This may partially be due to an underlying actin meshwork that acts as a fence together with some of the membrane proteins docked into this meshwork as pickets (hence, picket-fence model) (Jacobson et al., 1981; Dietrich et al., 2002; Fujiwara et al., 2002; Owen et al., 2009; Kusumi et al., 2010, 2011). Finally, owing to the development of superresolution optical microscopy, it is becoming clear that many membrane proteins are predominantly organized in nanometer-sized clusters and that multiple different clusters are co-existing in the plasma membrane (Sieber et al., 2007; Saka et al., 2014; Wilhelm et al., 2014).

Evidently, the presence of many different domains in the plasma membrane with distinct compositions cannot be explained by only one, or just a few, parameters. The plasma membrane contains thousands of different lipid and protein species (van Meer et al., 2008); hence a two-component system such as liquid ordered and liquid disordered phases does not provide a satisfactory explanation. Intriguingly, the protein occupancy of the bilayer volume (~20%) as indicated by the analysis of organelles (Takamori et al., 2006) and plasma membranes (Dupuy and Engelman, 2008) is much higher than originally anticipated. Thus, proteins may form phases on their own, together with certain lipids (Anderson and Jacobson, 2002). Additionally, one of the main characteristics of the plasma membrane is the asymmetry of the bilayer with the inner leaflet containing substantial amount of charged phospholipids (Bigay and Antonny, 2012). Therefore, new concepts are emerging, which consider electrostatic interactions, protein-protein (homo- and heterotypic) interactions, and hydrophobic interactions between bilayer core and protein transmembrane domains as parameters that contribute to segregation of proteins and lipids in distinct domains. Recently, a few studies have tried to merge these different mechanisms into a single model (Diaz-Rohrer et al., 2014; Milovanovic et al., 2015). In addition, it is becoming apparent that plasma membrane domains represent local hot spots that are essential for the functional segregation of distinct cellular processes. Due to technical limitations, progress in our understanding of membrane domains is largely confined to the plasma membrane. However, it will be interesting to understand if the same protein and lipid organizing principles also apply to organelles where they may play a role in processes such as sorting during trafficking and in organelle biogenesis.

Proteins involved in synaptic vesicle release have served as excellent models for analyzing the patterning of the plasma membrane. Synaptic vesicle release itself is a well-orchestrated process where a neurotransmitter-loaded vesicle attaches to the plasma membrane (a process known as docking), after which the fusion machinery enters a “preparatory” phase (known as priming) and then, once there is a calcium influx, the vesicle fuses with the plasma membrane (Südhof, 2004). Membrane fusion, the key step in neurotransmitter release is mediated by the interaction between protein members of the soluble NSF-attached protein receptor (SNARE) family that reside in the donor membrane with their cognate partners in the target membrane (Jahn and Scheller, 2006; Hong and Lev, 2014). SNARE proteins posses a central 60-70 AA-long motif (SNARE domain) that forms a coiled coil upon the interaction with the cognate SNARE partners. This coiled coil is connected by sixteen layers of interacting amino acid side chains that are hydrophobic (the flanking are all polar or charged) except of the amino acids in the central layer, which are either glutamine (Q) or arginine (R). Generally, the coiled-coil SNARE complex has three domains that contain glutamine together with one that contains arginine (QabcR) (Sutton et al., 1998; Antonin et al., 2002; Stein et al., 2009). The SNAREs involved in neuronal exocytosis include the plasma membrane residents syntaxin 1A (Qa) and SNAP 25 that contributes with two SNARE motifs (Qbc), and synaptobrevin 2 at the synaptic vesicle (R).

In recent years, an increasing body of evidence has revealed that SNAREs form clusters in both plasma membranes and intracellular membranes. Multiple approaches have yielded an increasingly refined picture of the forces and of the other biophysical parameters responsible for SNARE clustering, which will be discussed in the following chapters.

## Segregation within the hydrophoibc core of the membrane

### Clustering induced by lipid phases

As discussed above, lipid-based domain segregation was first postulated based on the observation that certain proteins tend to associate with specific lipid species (most conspicuously with cholesterol and sphingomyelin) and resist extraction by some nonionic detergents (Simons and van Meer, 1988; Brown and Rose, 1992; Schroeder et al., 1994). For many years, it has been controversially discussed whether these “detergent-resistant membranes (DRMs)” represent pre-existing structural entities that are preserved during extraction or are artificial aggregates generated by the extraction itself (Silvius et al., 1996; London and Brown, 2000; Heerklotz and Seelig, 2002; Sot et al., 2002; de Almeida et al., 2003). Presently, consensus is emerging that DRMs are largely created during extraction but many of the components enriched in DRMs appear to also associate with each other in an intact membrane. Thus, DRMs continue to be a useful tool for identifying candidates for membrane domains, but they do not represent a homogeneous population. For instance, GPI-anchored thymocyte antigen 1, ganglioside GM-1, and the membrane spanning linker for activation of T cells are all enriched when purified using DRMs, but are shown to form distinct clusters in the membrane (Wilson et al., 2004; Lichtenberg et al., 2005).

Although initial studies reported enrichment of SNAREs within DRMs (Lafont et al., 1999; Chamberlain et al., 2001; Chamberlain and Gould, 2002; Predescu et al., 2005; Salaün et al., 2005), it soon became clear that by applying different detergents, SNAREs were not co-floating with the classical DRM markers (Lang et al., 2001; Ohara-Imaizumi et al., 2004a). However, similar to DRMs cholesterol is required for the integrity of SNARE clusters (Lang et al., 2001; Lang, 2007). Furthermore, cholesterol depletion inhibits exocytosis in both neuronal (Lang et al., 2001) and non-neuronal cells such as epithelial (Chintagari et al., 2006) and endothelial (Predescu et al., 2005) cells, but it is still unclear whether dispersal of SNARE clusters and inhibition of fusion are causally related. Beyond neurotransmitter release, SNARE clusters are shown to be the release sites for cytokines at the phagocytic cup (Kay et al., 2006) and insulin (Ohara-Imaizumi et al., 2004a,b). Additionally, *in vitro* reconstitution of neuronal SNARE proteins into giant unilamellar liposomes capable of undergoing phase segregation suggested that SNAREs distribute in the liquid disordered phase (unsaturated phospholipids, cholesterol depleted regions), rather than in the liquid ordered phases (rich in saturated phospholipids and cholesterol). Although such simple phase-separation may not reflect phase-partitioning in the plasma membranes, these studies confirmed that SNAREs do not associate with sphingomyelin and saturated phospholipids (Saslowsky et al., 2003; Bacia et al., 2004). On the other hand they demonstrate that SNARE proteins are sensitive to such phase partitioning, raising the possibility that phase heterogeneity may contribute to SNARE segregation.

### Clustering induced by hydrophobic mismatch

Hydrophobic mismatch occurs when the length of the protein transmembrane domains (TMDs) does not match the bilayer thickness. In this case, it is energetically favorable to cluster the TMDs of similar length in the same region rather than to accommodate each of the TMDs separately. In a theoretical paper, Mouritsen and Bloom proposed that proteins may cluster in order to minimize membrane mismatch (Mouritsen and Bloom, 1984). Pioneering research showed that certain enzymes have the highest activity when reconstituted in bilayers of particular thickness, whereas in both thinner and thicker bilayers the activity drops (Johannsson et al., 1981a,b; Kusumi and Hyde, 1982). This implied that hydrophobic mismatch affects enzyme conformation that subsequently reduces its activity. Moreover, the aggregation state of some of these proteins such as rhodopsin is shown to depend on the acyl-chain length of lipids that were used for the reconstitution (Kusumi and Hyde, 1982). More recently, it was also shown that the overlap between the TMD length of the perfringolysin O, a multispanning barrel protein, and the width of lipid bilayer also affects the proteins' distribution and functionality in proteoliposomes (Lin and London, 2013). Protein clustering driven by hydrophobic mismatch was first shown directly for synthetic TMD peptides (de Planque et al., 1998; Sparr et al., 2005). The phospholipid acyl-chains are flexible and their lateral organization depends on the neighboring lipid molecules (i.e., cholesterol restricts the flexibility due to the pronounced hydrophobic planar structure). Hence, lipids can adopt to a range of different thicknesses (Zaccai, 2000). On the other hand, proteins exhibit less flexibility in length distortion in the case of membrane mismatch (Petrache et al., 2002). Caution is needed when interpreting experiments based on altering acyl chain lengths because these changes also affect the lipid packing, curvature and surface charge distribution. Hence, the protein function may be affected by many of these parameters (Anderson and Jacobson, 2002).

Hydrophobic mismatch appears to play a role in defining the final destination of membrane components in intracellular trafficking. It is well-established that sorting of proteins and lipids in polarized, epithelial cells is mediated by both the lipid environment and the cytoskeleton, and that lipid domains coalesce prior to vesicle formation (Brown and Rose, 1992; Lipowsky, 1993; Yoshimori et al., 1996; Roux et al., 2005). Additionally, altering the TMD length of peptides affected their trafficking from ER, Golgi to the plasma membrane (Munro, 1991, 1995; Pelham and Munro, 1993; Nilsson et al., 1996). Considering that the average thickness of the membrane increases from ER (~3.75 nm) to the plasma membrane (~4.25 nm; Mitra et al., 2004), it is reasonable to expect that proteins destined to the plasma membrane have longer TMDs. Indeed, in a comprehensive screen of the TMDs sequences from different species, Sharpe et al. demonstrate that an average length of TMDs is about 5 amino acids shorter for proteins destined to ER compared to the proteins of the plasma membrane (Sharpe et al., 2010).

**Figure 1:**
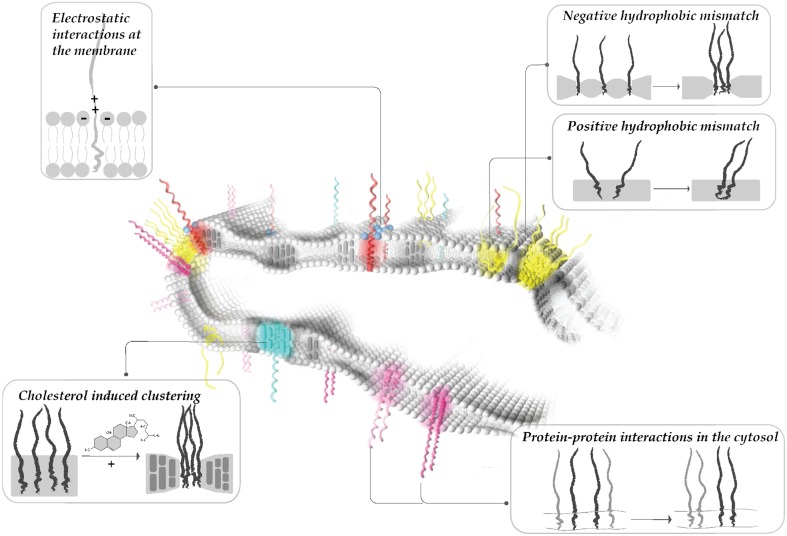
**Modularity of protein clustering**. Hydrophobic interactions between the core of the lipid bilayer and the protein TMDs (i.e., hydrophobic mismatch, interactions mediated by cholesterol), electrostatic interactions at the membrane surface (i.e., positive patches of proteins and polyphosphoinositides) and specific protein-protein interactions at the cytoplasmic surface all affect membrane patterning. Multiple mechanisms affect the protein/lipid organization simultaneously, leading to dynamic reorganization of protein clusters.

Intriguingly, thickness differences within the plane of the plasma membrane have recently been shown to be important for lateral sorting of proteins into distinct domains (Milovanovic et al., 2015). The crystal structure of the neuronal SNARE complex revealed that the TMD of syntaxin 1 might be too short to span the entire plasma membrane (Stein et al., 2009). In comparison to syntaxin 1 (involved in calcium regulated exocytosis), syntaxin 4 (involved in the constitutive exocytosis) has a slightly (1–2 residues) longer TMD. We have recently shown that the TMD length difference of a single amino acid between syntaxin 1 and syntaxin 4 can drive the proteins into separate clusters in the plasma membrane (Milovanovic et al., 2015). Here, the effect of cholesterol on local alterations of membrane thickness plays an important role. The average plasma membrane thickness is estimated to be around 4 nm (Mitra et al., 2004) and incorporation of physiological amounts (30%) of cholesterol increases the membrane thickness by about 0.35 nm (Milovanovic et al., 2015). Therefore, cholesterol can drive protein clustering by inducing or enhancing local hydrophobic mismatch in the membrane (Figure 1). This probably explains why cholesterol depletion causes the dispersal of SNARE domains in the membrane and a concomitant decrease in exocytosis (Lang et al., 2001; Chamberlain and Gould, 2002; Ohara-Imaizumi et al., 2004a; Kay et al., 2006).

### Palmitoylation modulates attachment of soluble proteins to the membrane

Posttranslational modifications further modulate SNARE patterning (for detailed review see van den Bogaart et al., 2013). Most attention has been paid to palmitoylation, i.e., the covalent addition of the acyl chain palmitate (C16:0) to a cysteine residue in the protein. For instance, the Qbc SNAREs SNAP 23 and 25 are palmitoylated (Prescott et al., 2009) at five and four cysteine residues, respectively, which is required for membrane attachment. Proteomics analyses suggested that many other synaptic proteins undergo palmitoylation including proteins containing TMDs (Kang et al., 2008), among these are the SNAREs syntaxin 1 and synaptobrevin 2. It has been suggested that SNAREs are reversibly targeted to cholesterol and sphingomyelin rich regions via palmitoylation (Fukata and Fukata, 2010; Levental et al., 2010), which would add another mechanism contributing to cluster formation. Support for this concept is provided by the recent finding that a fraction of amyloid precursor is palmitoylated, which further modulates its association with cholesterol-rich regions in the presynaptic membrane (Bhattacharyya et al., 2013).

## Segregation caused by interactions at the hydrophobic-hydrophilic boundary

Clustering of SNAREs is influenced by electrostatic interactions between positively charged side chains adjacent to the hydrophobic TMD with negatively charged polyphosphoinositides (Di Paolo et al., 2004; Do Heo et al., 2006; van den Bogaart et al., 2011). Tamm and colleagues (Wagner and Tamm, 2001) showed that diffusion of syntaxin 1 decreases upon incorporation of PI(4,5)P_2_ in the lipid monolayer. PI(4,5)P_2_ is enriched in regions of the plasma membrane where secretory vesicles dock (Laux et al., 2000; Aoyagi et al., 2005), and it is essential for exocytosis (Hay and Martin, 1993; Milosevic et al., 2005; James et al., 2008; Wen et al., 2011). Although PI(4,5)P_2_ comprises only 1% of total lipids of the plasma membrane (Di Paolo and De Camilli, 2006), it can reach concentrations of more than 80% of total lipids in clusters (van den Bogaart et al., 2011). Association between syntaxin 1 and PI(4,5)P_2_ was clearly shown both *in vitro* reconstituted systems (Murray and Tamm, 2009, 2011) and in cells (van den Bogaart et al., 2011). Responsible for this strong interaction is a cluster of positively charged arginines and lysines directly adjacent to the TMD of syntaxin 1 (van den Bogaart et al., 2011; Khuong et al., 2013).

Ionic interactions between macromolecules are strongly influenced by mobile ions. The ionic composition at the surface of a membrane is highly complex (Wang et al., 2012, 2014), rendering it difficult to quantify the influence of ions on domain formation. Ions present at high concentrations on the cytoplasmic surface (K^+^, Mg^2+^, glutamate, ATP) are able to shield the charge of both lipid head-groups and proteins involved in exocytosis (Park et al., 2012). It is worth noting that calcium increases syntaxin 1 clustering in the plasma membrane of PC12 cells (Zilly et al., 2011), and this mechanism might involve the interaction with negatively charged lipids. Polybasic clusters on the cytoplasmic face adjacent to transmembrane proteins are common among many membrane proteins (von Heijne, 2006). Thus, it is possible that such ionic interactions play a major role in patterning of the plasma membrane and possibly also of intracellular membranes (Figure 1).

## Segregation due to interactions in the hydrophilic space

Both homophilic and heterophilic interactions have been described for Qa SNARE family members. For instance, syntaxin 1 and syntaxin 4 are involved in regulated and constitutive exocytosis, respectively. Interactions between the SNARE motifs at the cytoplasmic surface has been suggested to contribute to the segregation of these proteins into distinct domains (Sieber et al., 2006). Hence, in case of syntaxin isoforms homotypic protein interactions contribute to the functional segregation (Figure 1). Similarly, in non-neuronal cells, syntaxin isoforms segregate in different regions of the membrane. In highly polarized epithelial cells, syntaxin 3, and syntaxin 4 are trafficked distinctly to the apical and basolateral membrane, respectively. Even the deletion of the targeting signal of syntaxin 3 does not eliminate its distinct segregation from syntaxin 4 enriched regions (Low et al., 2006).

Heterotypic protein interactions are important for both the spatial sorting of proteins in the presynapse, as well as for catalyzing the fusion reaction. For instance, some presynaptic membrane proteins bind to the actin meshwork (Torregrosa-Hetland et al., 2011, 2013; Villanueva et al., 2012). This binding to the cytoskeleton can be direct as in the case of syntaxin 4 (Jewell et al., 2008; Woronowicz et al., 2010) and SNAP 25 (Torregrosa-Hetland et al., 2013). Alternatively, binding to actin can be mediated by adaptor proteins such as myosin V that connects syntaxin 1 to actin (Watanabe et al., 2005), and α-fodrin that connects syntaxins 3 and 4 to actin (Nakano et al., 2001). Another example for heterotypic interactions includes binding of regulatory proteins to SNAREs. The SM-protein Munc 18 that binds to syntaxin 1 is not only essential for exocytosis (Verhage et al., 2000) but also necessary for trafficking of syntaxin 1 to the plasma membrane (Voets et al., 2001; Yang et al., 2006; Kurps and de Wit, 2012). Indeed, if syntaxin 1 clusters serve as reservoir of the protein for fusion, Munc 18 may be needed to pry a individual syntaxin 1 molecules away from the cluster (Bar-On et al., 2012). Munc 18 bound syntaxin 1 is able to recruit SNAP 25 in the cell lawns and synaptobrevin 2 containing vesicles can bind to this complex (Zilly et al., 2006).

## Functional relevance of SNARE clustering

### SNARE clustering may be important for exocytosis

**Figure 2:**
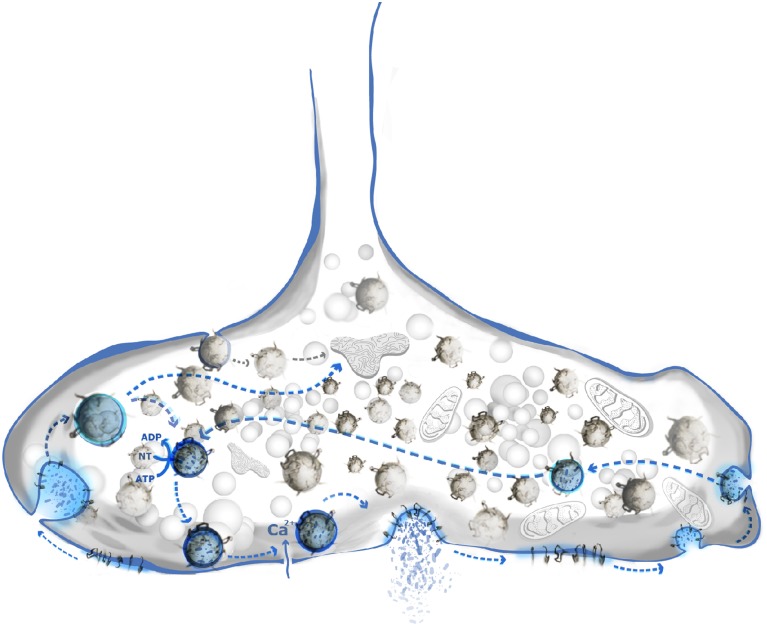
**SNARE clustering has multiple roles in synaptic vesicle cycle**. Classical synaptic vesicle cycle (blue shade) includes: neurotransmitter loading, docking, priming, calcium triggered release, subsequent engulfing, and recycling of vesicle. Syntaxin 1/PI(4,5)P_2_ domains are shown to play an important role in vesicle docking. Components of vesicle's membrane remain clustered or are re-clustered prior to endocytosis. SNARE domains may be a sorting determinant, especially in the case of vesicles engulfed by rapid endocytosis.

Several lines of evidence suggest three important physiological roles for SNARE clustering in exocytosis (Figure 2). First, the high local concentrations of SNAREs at the plasma membrane may provide the functional pools of proteins necessary for the formation of SNARE complexes. Clustering of SNAREs may also prevent nonproductive side-reactions of the highly reactive SANREs such as the formation of so-called “dead-end” complexes between syntaxin 1 and SNAP 25 incapable of fusion (Fasshauer and Margittai, 2004). It has been shown that the plasma membrane of chromaffin cells lacks these dead-end complexes (Halemani et al., 2010). Also, removal of cholesterol does not only affect the clustering of SNAREs, but also reduces the number of functionally active syntaxin 1/SNAP 25 complexes ready for ternary complex formation with synaptobrevin 2 (Rickman et al., 2010). Second, Q-SNARE domains [together with PI(4,5)P_2_] may represent docking platforms for vesicles (James et al., 2008; de Wit et al., 2009; Imig et al., 2014). PI(4,5)P_2_ was shown to be enriched at the sites of vesicle fusion, and altering the amount of PI(4,5)P_2_ affects the release capacities (Milosevic et al., 2005; de Wit et al., 2009). Therefore, PI(4,5)P_2_ domains have been proposed to act as molecular beacons for vesicle recruitment to the membrane. Indeed, synaptotagmin 1, the main calcium sensor for exocytosis, binds to the syntaxin 1/PI(4,5)P_2_ clusters in the plasma domains (Honigmann et al., 2013). Finally, clustering may decrease the energy barrier that needs to be overcome for membrane fusion in two ways. First, line tension around the cluster locally destabilizes the membrane. Based on several studies, such micro-destabilized regions decrease the energy barrier needed for membrane remodeling (Boucrot et al., 2012; Kozlov et al., 2014; Risselada et al., 2014). The lower the energy barrier, the less SNARE complexes are needed for successful fusion (Mohrmann et al., 2010; van den Bogaart et al., 2010; Hernandez et al., 2014). Secondly, initial experiments emphasized that the interaction of not only SNARE motifs but also of the C-terminal TMDs of syntaxin 1 and synaptobrevin 2 was seen as necessary for the processing from the hemifused to the fully fused state (i.e., fusion pore opening) (Grote et al., 2000; Han et al., 2004; Fdez et al., 2010). Recent work from Südhof's lab indicated that this is not the case since fusion was possible in cells where the syntaxin 1 TMD was replaced with a lipid anchor (Zhou et al., 2013). Interestingly, bioactive molecules such as anesthetics (e.g., isoflurane, etomidate and propofol) reduce the release capacities of chromaffin cells (Herring et al., 2009, 2011; Xie et al., 2013). Since they interact with SANREs and SNARE-associated proteins, they may be altering the lateral organization of SNARE clusters (Herring et al., 2011). Clustered SNAREs are in dynamic exchange with the surrounding membrane, and diffusion of SNARE molecules between clusters is rather high (Sieber et al., 2007; Barg et al., 2010; Knowles et al., 2010; Wilhelm et al., 2014). As discussed above, syntaxin clusters may serve as molecular beacons (or hot spots) for vesicle docking. However, the exact assembly of fusion competent SNARE complexes most probably takes place adjacent to these clusters (Rickman et al., 2005; Bar-On et al., 2012; Gandasi and Barg, 2014).

### SNARE clustering may be important for endocytotic retrieval of vesicles

Neurotransmitter release is a rapid and repetitive process. In order to maintain membrane balance vesicle fusion and fission have to be tightly spatially and temporally coupled (Figure 2). During endocytosis, vesicle-specific proteins are selectively retrieved while plasma membrane residents are excluded. Even during sustained, high activity, the composition of synaptic vesicles needs to remain constant. While some flexibility may be tolerated for abundant proteins such as synaptobrevin 2 (~70 copies/vesicle) some of the functionally essential proteins are present only 1–2 copies/vesicle, e.g., the vacuolar ATPase required for neurotransmitter uptake (Takamori et al., 2006). Using STED microscopy, Willig et al. (2006) suggested that that SV proteins remain clustered after exocytosis. Alternatively, SV proteins may be sorted and re-clustered prior to endocytosis (Hua et al., 2011). Further, SNAREs are connected to the proteins that regulate the vesicle release such as soluble SM proteins and tethering factors. The region of the presynaptic membrane where vesicles dock and fuse is distinguished by defined structural elements (Szule et al., 2012; Fernandez-Busnadiego et al., 2013; Harlow et al., 2013; Imig et al., 2014). These “active zones” contain multiple protein complexes that regulate tethering and docking (Fernandez-Busnadiego et al., 2013; Imig et al., 2014), but the details of the structure and dynamics of the underlying protein-protein interactions are only slowly emerging. Interestingly, a study that combined the electron microscopy and the STED nanoscopy showed that synaptic vesicle proteins such as synaptotagmin remain clustered even within the early endosome, thus being a marker for synaptic vesicle retrieval (Hoopmann et al., 2010). Generally, there appear to be at least two main pathways for vesicle endocytosis: (i) slow, clathrin-mediated endocytosis (CME), and (ii) fast, mostly clathrin-independent, endocytosis. CME has been extensively studied (Jung and Haucke, 2007; Dittman and Ryan, 2009). The relatively slow kinetics of CME (~20 s) cannot fully explain fast vesicle turnover at the synaptic bouton (Heuser and Reese, 1973; Gandhi and Stevens, 2003). Using a combination of optogenetics and high-pressure freezing electron microscopy, Jorgensen and colleagues showed that a second type of endocytosis co-exists in neurons that can be very rapid (~30 ms) but is likely to be less accurate than CME, resulting in endocytotic membrane vesicles larger than SV (Watanabe et al., 2014a,b, 2013a,b). Apart from speed, the availability of endocytotic machinery might be the limiting step in CME during the sustained SV release. Indeed, quantitative analysis of the synaptic bouton showed that there are about five folds less endocytotic than exocytotic proteins (Wilhelm et al., 2014). This problem may be overcome by fast, bulk endocytosis that requires fewer proteins to be involved in vesicle engulfing (Watanabe et al., 2013b).

It is still debated to which extent endocytosed vesicles need to pass through an additional endosomal sorting step before re-entering the SV pool. It is conceivable that the fate of the endocytosed membrane is determined by its protein and lipid components (Rizzoli, 2014). Shortly after exocytosis the protein content of the synaptic vesicle either remains clustered (Willig et al., 2006), or it diffuses in the plane of the membrane which is followed by immediate re-clustering (Wienisch and Klingauf, 2006; Hua et al., 2011). Specific adaptor proteins such as AP2, stonin and AP 180 specifically bind to synaptic vesicle proteins such as synaptobrevin 2 (AP 180) (Granseth et al., 2006) or synaptotagmin 1 (AP2, stonin) (Collins et al., 2002; Jung et al., 2007), ensuring their clustering in a coated pit (Glyvuk et al., 2010). It is conceivable that a clathrin-coated vesicle separating from the plasma membrane matches the membrane composition of synaptic vesicles (as already suggested earlier; Maycox et al., 1992), allowing for immediate re-use after uncoating without an intermediate sorting step (Watanabe et al., 2014b). In contrast, it is highly unlikely that vesicles retrieved from the plasma membrane by ultrarapid endocytosis are sorted with similarly high accuracy. It is conceivable that these vesicles need to “proof-read” by cytoplasmic factors after endocytosis (Figure 2). If the protein and lipid content of such an endocytosed vesicle meets the requirements for a functional synaptic vesicle, the vesicle might be loaded with NT and can be immediately used for the next round of the release. Otherwise, the vesicle is targeted to recycling endosomes for further sorting. The precise sorting mechanism is far from understood and the sorting signals involved in vesicle recycling and the maintenance of the vesicle identity still need to be identified.

## Conclusion

SNARE proteins have turned out to be excellent paradigms for studying the biochemical and biophysical mechanisms that govern domain formation in biological membranes. The picture that is emerging reveals a modular interplay of different forces and interactions that are all required for the complex patterning of a membrane into multiple different clusters (Figure 1). These parameters include: (i) hydrophobic interactions between the core of the lipid bilayer and the hydrophobic membrane anchors of the proteins such as hydrophobic mismatch, phase partitioning, and cholesterol-mediated interactions, (ii) interactions between negatively charged lipid head-groups with positively charged clusters frequently observed at the membrane surface, (iii) specific protein–protein interactions at the cytoplasmic surface. Many of these clusters are not static features of the membrane but rather represent dynamic entities where clustered and thus mostly immobile proteins and lipids are in equilibrium with their rapidly diffusing monomeric or oligomeric counterparts. Future work is needed to appreciate the heterogeneity of the clusters, their molecular composition, and to clarify whether only some or most membrane proteins are organized in such domains.

### Conflict of interest statement

The authors declare that the research was conducted in the absence of any commercial or financial relationships that could be construed as a potential conflict of interest.
